# Lazy Adrenals in Severe Hypothyroidism - Myth or Mirage?

**DOI:** 10.5812/ijem-158985

**Published:** 2025-04-30

**Authors:** Swaraj Waddankeri, Meenakshi Swaraj Waddankeri

**Affiliations:** 1 Mahadevappa Rampure Medical College, Kalaburagi, India; 2 Department of Paediatrics, M R Medical College, Kalaburagi, India

**Keywords:** Hypothyroidism, Adrenal Insufficiency, Cortisol, Thyroid-Stimulating Hormone, Triiodothyronine, Thyroxine

## Abstract

**Background:**

Clinical features of hypothyroidism and adrenal insufficiency (AI) often overlap.

**Objectives:**

To assess the morning serum cortisol levels of treatment-naïve patients with severe hypothyroidism.

**Methods:**

In this prospective, case-control study, treatment-naïve adults with severe hypothyroidism [thyroid-stimulating hormone (TSH) > 100 mIU/mL] were compared with age- and sex-matched euthyroid controls. Morning (08:00 AM) serum cortisol, TSH, triiodothyronine (T3), and thyroxine (T4) levels were measured. AI was defined clinically and biochemically as cortisol levels < 4 µg/dL. Correlation coefficients between T3, T4, and cortisol levels were calculated.

**Results:**

The case group (n = 71; women, 88.7%; mean age, 30.0 ± 9.0 years) had significantly lower serum cortisol levels than controls (n = 40; 8.6 ± 4.2 vs 16.0 ± 2.22 µg/dL; P < 0.0001). Six patients (8.5%) in the case group met criteria for AI. Patients with AI had significantly lower T3 and T4 levels than those without AI (P = 0.018 and P = 0.005, respectively). A negative correlation was observed between T3 and cortisol levels (r = -0.243, P = 0.041), while T4 showed no significant correlation (r = -0.103, P = 0.391).

**Conclusions:**

Treatment-naïve patients with severe hypothyroidism may exhibit biochemical evidence of AI. Routine screening for AI in these patients is recommended to avoid missed diagnoses and guide appropriate therapy.

## 1. Background

Thyroid disorders, such as hypothyroidism and hyperthyroidism, are among the most routinely encountered endocrinopathies worldwide. Their prevalence varies geographically, and hypothyroidism is generally more common than hyperthyroidism (1). In India, the prevalence of hypothyroidism in the adult population is nearly 11% (2). Owing to advances in the diagnosis of thyroid abnormalities and increased availability of testing, severe primary hypothyroidism has become a rare observation (3-6). However, hypothyroidism may remain undiagnosed due to nonspecific symptoms and a lack of awareness (7), eventually leading to severe hypothyroidism, which may manifest as myxedema coma (8). Further, cortisol production and its utilization are reduced in severe hypothyroidism (9), which may be why glucocorticoid therapy (e.g., hydrocortisone 50 - 100 mg IV every 8 hours) is administered to patients with myxedema before initiating levothyroxine (10). The features of hypothyroidism and associated adrenal insufficiency )AI) often overlap in clinical settings, resulting in the attribution of symptoms to hypothyroidism rather than to AI. Therefore, assessment of AI in patients with severe hypothyroidism is essential. Measurement of morning serum cortisol levels is a simple and easy method of diagnosing AI (indicated by cortisol levels < 4 µg/dL) (11-13).

## 2. Objectives

We aimed to assess the morning serum cortisol levels of treatment-naïve patients with severe hypothyroidism [thyroid-stimulating hormone (TSH) levels > 100 mIU/mL] at first presentation.

## 3. Methods

### 3.1. Design and Setting

This prospective, observational, case-control, propensity-matched study was conducted at a tertiary care teaching hospital in Kalaburagi, Karnataka, India.

### 3.2. Participants

We included consecutive treatment-naïve patients (age, 18 - 50 years) with severe hypothyroidism, who were referred from the Division of Diabetes and Endocrinology, Department of Internal Medicine, between January 2017 and March 2020. All patients (71 treatment-naïve adults) were diagnosed with severe hypothyroidism based on TSH levels of > 100 mIU/mL. We excluded patients with a history of Grave’s disease, pituitary or adrenal disorder, thyroidectomy, or intake of anti-thyroid drugs, radioactive iodine, and steroids; pregnant patients; patients on oral contraceptives; and those with underlying malignancies. Individuals visiting the Department of Internal Medicine without thyroid disease or any comorbidity that could affect the hypothalamo-pituitary-adrenal axis function were included as controls. The hypothyroidism and control groups were age- and sex-matched.

### 3.3. Materials and Methods

The clinical features (symptoms such as fatigue, hypotension, and salt craving were considered) of all participants were examined by a single investigator and recorded on a pre-designed proforma. These features included Zulewski’s clinical score (14), anthropometric measurements, presence of goiter, and its World Health Organization grading (15). The participants’ serum triiodothyronine (T3), thyroxine (T4), TSH, and cortisol levels were analyzed using a fully automated immunoassay analyzer (AIA360^®^, Tosoh Bioscience) and commercially available kits in accordance with the manufacturers’ instructions. For TSH, the intra-assay (within run) precision was 2.3%, and the inter-assay (between run) precision coefficient of variation (COV) was 3.3%. For T4, the intra-assay (within run) precision was 3%, and the inter-assay (between run) precision COV was 4.4%. For T3, the intra-assay (within run) precision was 3.7%, and the inter-assay (between run) precision COV was 3.2%. For cortisol, the intra-assay (within run) precision was 2.5%, and the inter-assay (between run) precision COV was 4.7%.

For cortisol estimation, a fasting sample was obtained from all participants between 8:00 - 9:00 AM. Based on cortisol levels, the participants were categorized into three groups: ≤ 4 µg/dL, 4.1 – 9 µg/dL, and > 9 µg/dL.

#### 3.3.1. Inclusion Criteria

Adults aged 18 - 50 years. Treatment-naïve patients diagnosed with severe hypothyroidism, defined by TSH > 100 mIU/mL.

#### 3.3.2. Exclusion Criteria

History of Graves’ disease. Known pituitary or adrenal disorders. Thyroidectomy. Use of: Anti-thyroid drugs, radioactive iodine, steroids, oral contraceptives. Pregnancy. Underlying malignancies.

### 3.4. Statistical Analyses

The data were analyzed using SAS 9.4 software (SAS Institute, Cary, NC). The serum T3, T4, and cortisol levels were categorized and summarized descriptively. Continuous variables are presented as mean and standard deviation, and categorical parameters as counts and percentages. Correlation coefficients for serum T3, T4, and cortisol levels were generated for the overall study population. A P-value of < 0.05 was considered to indicate statistical significance. This analysis was powered by Medeva (https://medeva.io), an analytics-embedded electronic health record platform.

### 3.5. Ethical Aspects

The study was approved by the institutional ethics committee on February 13, 2021. Written informed consent was obtained for participation in the study and use of the patient data for research and educational purposes. The procedures in the study follow the guidelines laid down in the Declaration of Helsinki.

## 4. Results

Seventy-one patients with severe hypothyroidism [defined as a state with a TSH level > 100 mIU/mL in a treatment-naïve individual, often associated with marked symptoms and biochemical evidence of thyroid hormone deficiency low T3 and T4 levels)] were enrolled in the case group (mean age, 30.0 ± 9.0 years; women, 88.7%). Most of them complained of fatigue (n = 64; 90.1%), followed by excess hair fall (n = 36; 50.7%), oligomenorrhea (n = 30; 42.3%), and discomfort (n = 27; 38.0%). None of the participants had symptoms suggestive of low cortisol levels or AI. Their mean T3 and T4 levels were 34.35 ± 31.85 ng/dL and 1.91 ± 1.38 µg/dL, respectively. In addition, 42 (59.2%) patients had serum T3 levels of ≤ 40 ng/dL, and 55 (77.5%) had serum T4 levels of < 3 µg/dL. The characteristics of the case group are presented in Tables 1 and 2. 

**Table 1.** Comparison of Demographic, Clinical, and Biochemical Characteristics Between Case and Control Groups ^a^

Parameters	Case Group (n = 71)	Control Group (n = 40)	P-Value
**Age (y)**	30.0 ± 9.0	27.9 ± 6.9	0.215
**Gender**			0.979
Female	63 (88.7)	36 (90.0)	
Male	8 (11.3)	4 (10.0)	
**BMI (kg/m** ^**2**^ **)**	23.9 ± 4.6	-	-
**Clinical Presentation**		-	-
Fatigue	64 (90.1)	-	-
Excess hair fall	36 (50.7)	-	-
Oligomenorrhea	30 (42.3)	-	-
Discomfort	27 (38.0)	-	-
Proximal muscle weakness	24 (33.8)	-	-
Excess tiredness	24 (33.8)	-	-
Throat pain	22 (31.0)	-	-
Difficulty in swallowing	17 (23.9)	-	-
Vitals		-	-
Systolic BP (mmHg)	114.4 ± 13.2	-	-
Diastolic BP (mmHg)	75.2 ± 11.6	-	-
Pulse rate (bpm)	65.3 ± 7.1	-	-
**Biochemical Parameters**			
TSH (mIU/mL)	100.0 ± 0.0	2.35 ± 0.78	< 0.0001
Cortisol (µg/dL)	8.6 ± 4.2	16.0 ± 2.22	< 0.0001

Abbreviation: BMI, Body Mass Index.

^a^ Values are expressed as mean ± SD or No. (%).

**Table 2.** Zulewski’s Score, Goiter Grade, and Thyroid Hormone Levels of Patients with Severe Hypothyroidism ^a^

Parameters	Finding
**Zulewski’s score**	6.4 ± 1.7
≥ 5	59 (83.1)
3 - 5	12 (16.9)
< 3	0
**Goiter grade**	
Grade 0	16 (22.5)
Grade 1	1 (1.4)
Grade 1	6 (8.5)
Grade 2	26 (36.6)
Grade 3	22 (31.0)
**Thyroid hormone levels**	
T3 (ng/dL)	34.35 ± 31.85
≤ 40	42 (59.2)
> 40	29 (40.8)
T4 (µg/dL)	1.91 ± 1.38
< 3	55 (77.5)
> 3	16 (22.5)
**Serum cortisol (µg/dL)**	8.58 ± 4.18

Abbreviations: T3, triiodothyronine; T4, thyroxine; TSH, thyroid-stimulating hormone.

^a^ Values are expressed as mean ± SD or No. (%).

**Figure 1.**
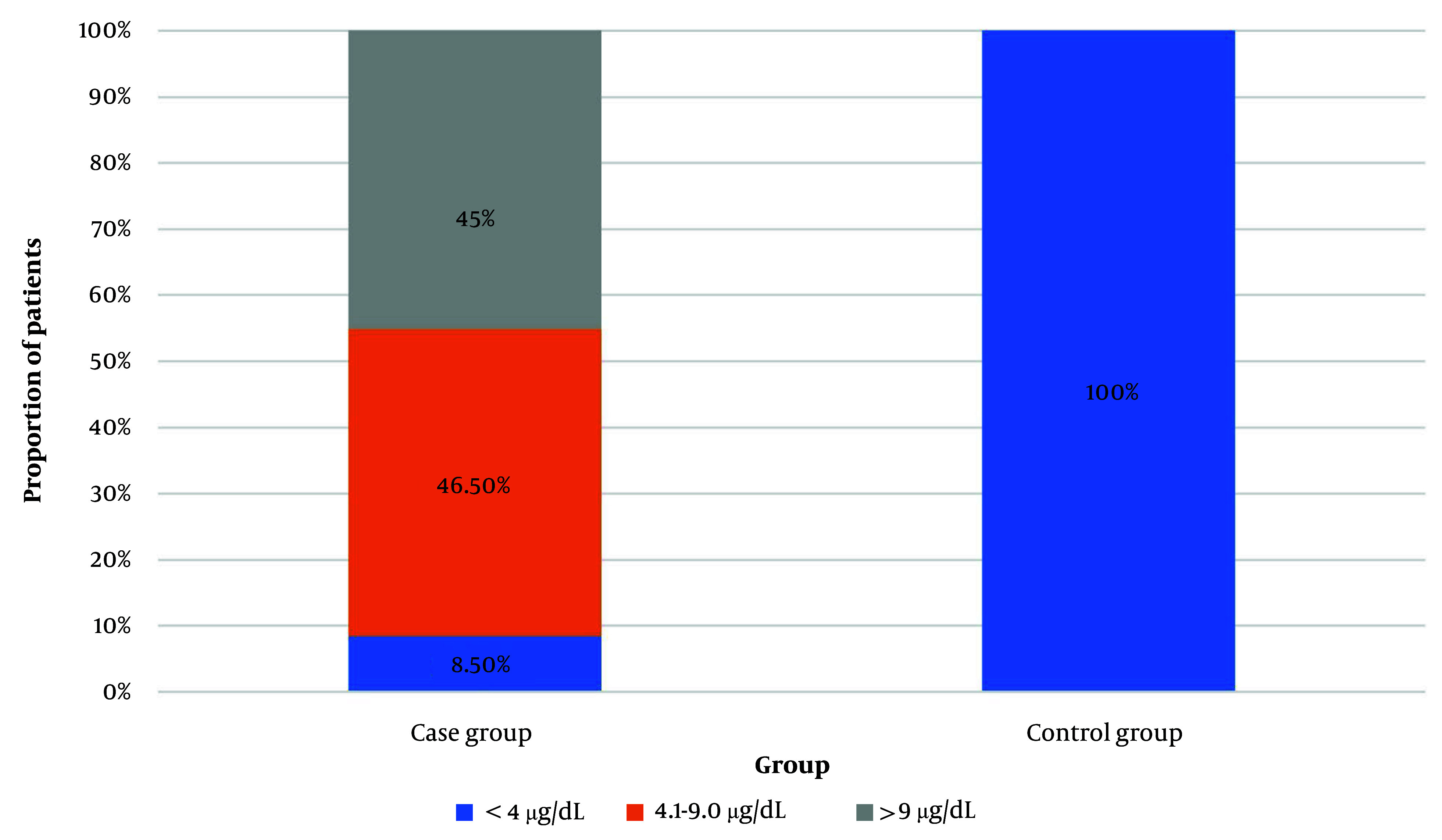
Distribution of the cortisol level groups across the case and control group

The data of the case group were compared with those of 40 age- and sex-matched controls (Table 1). The mean TSH level of the control group was significantly lower than that of the case group (2.35 ± 0.78 mIU/mL; P < 0.0001), and the mean serum cortisol level was significantly higher in controls than in the case group (16.0 ± 2.22 vs 8.6 ± 4.2 µg/dL, respectively; P < 0.0001). The participants were categorized into three groups based on their cortisol levels (≤ 4 µg/dL, 4.1 - 9 µg/dL, and > 9 µg/dL) (Figure 1). In the case group, six (8.5%), 33 (46.5%), and 32 (45%) patients had serum cortisol levels of ≤ 4 µg/dL, 4.1 – 9 µg/dL, and > 9 µg/dL, respectively. On the other hand, all controls had serum cortisol levels of > 9 µg/dL. The mean T3 and T4 levels of the three cortisol groups did not differ significantly (Table 3). However, there was a significant negative correlation between serum T3 and cortisol levels (r = -0.243, P = 0.041). Although the serum T4 and cortisol levels exhibited a negative correlation, it was not statistically significant (r = -0.103, P = 0.391) (Table 3). 

**Table 3.** Association Between the Cortisol and Thyroid Hormone Levels in the Case Group ^a^

Thyroid Hormones	Cortisol ≤ 4 µg/dL (n = 6)	Cortisol 4.1 - 9 µg/dL (n = 33)	Cortisol > 9 µg/dL (n = 32)	P-Value
**T3 levels (ng/dL)**	71.5 ± 28.1	36.5 ± 30.2	29.8 ± 32.0	0.158 ^b^ / 0.072 ^c^
**T3 group (≤ 40 ng/dL)**	0 (0)	17 (40.5)	25 (59.5)	0.078 ^d^
**T3 group (> 40 ng/dL)**	3 (10.4)	13 (44.8)	13 (44.8)	-
**T4 levels (µg/dL)**	3.1 ± 0.7	1.8 ± 1.3	1.9 ± 1.5	0.295 ^b^ / 0.459 ^c^
**T4 group (≤ 3 µg/dL)**	1 (1.9)	25 (45.5)	29 (52.8)	0.138 ^d^
**T4 group (> 3 µg/dL)**	2 (12.5)	5 (31.3)	9 (56.3)	-

Abbreviations: T3, triiodothyronine; T4, thyroxine; TSH, thyroid-stimulating hormone.

^a^ Values are expressed as mean ± SD or No. (%).

^b^ P-value from Kruskal-Wallis test.

^c^ P-value from Jonckheere-Terpstra trend test.

^d^ P-value from chi-square test.

The serum T3 and T4 levels of the case-group patients with and without AI are presented in Table 4. The case-group patients without AI had significantly higher mean serum T3 (39.33 ± 38.92 vs 20.32 ± 16.94 ng/dL, P = 0.018) and T4 (1.93 ± 1.17 vs 0.81 ± 0.55 µg/dL, P = 0.005) levels than those of six propensity-matched [based on age, Body Mass Index (BMI), and Zulewski’s score] patients with AI. To eliminate confounding factors such as age, BMI, and severity of hypothyroidism (assessed using Zulewski’s score), a matched subgroup analysis was performed. Six patients without AI were selected to match the AI group (n = 6) on these parameters. Even after matching, the non-AI group maintained significantly higher serum T3 (39.33 ± 38.92 ng/dL vs 20.32 ± 16.94 ng/dL) and T4 (1.93 ± 1.17 µg/dL vs 0.81 ± 0.55 µg/dL) levels than their AI counterparts.

**Table 4.** Thyroid Hormone Levels in Cases with and Without Adrenal Insufficiency ^a^

Parameters	No AI (n = 65) ^b^	No AI (n = 6) ^c^	AI (n = 6)	P-Value
**T3 (ng/dL)**	35.56 ± 32.67	39.33 ± 38.92	20.32 ± 16.94	0.018
**T4 (µg/dL)**	2.01 ± 1.39	1.93 ± 1.17	0.81 ± 0.55	0.005

Abbreviations: T3, triiodothyronine; T4, thyroxine; AI, adrenal insufficiency.

^a^ Values are expressed as mean ± SD.

^b^ Without matching.

^c^ Matched for age, Body Mass Index, and Zulewski’ score.

## 5. Discussion

We observed a significant negative correlation between serum T3 and cortisol levels in this study, which was expected as T3 levels are lower in severe hypothyroidism. The T3 and T4 levels of cases without AI were higher than those in propensity-matched cases with AI. This finding raises two important questions. The first question is whether the reduction in T3 and T4 levels is more pronounced in patients with AI, and the second is whether this is a physiological or pathological response with implications for treatment planning. A possible explanation for this finding is the altered metabolism of thyroid hormones. Kasperlik-Załuska et al. found that of 24 patients with unexplained AI, 14 had primary hypothyroidism and 23 exhibited antibodies against peroxidases (16). This indicates a potential association between autoimmune thyroid dysfunction and AI. However, clinical prediction of AI in severe hypothyroidism remains challenging, and further research on this aspect is necessary.

Patients with TSH levels of > 100 mIU/mL are rarely encountered in the Western world, which may be attributed to the easy access to high-quality TSH testing, extensive screening programs, and the lower clinical threshold to prescribe TSH testing. However, hypothyroidism may sometimes go unnoticed due to nonspecific symptoms and lead to severe hypothyroidism (TSH levels > 100 mIU/mL) (7, 8). Over the study duration (≈ 3 years), we identified 71 patients with severe hypothyroidism (TSH > 100 mIU/mL), who sought medical care at our institution on an outpatient basis.

Severe hypothyroidism is associated with an increased risk of myxedema coma and organ dysfunction of varying severity. Dutta et al. studied patients with myxedema coma and found that 39% of them were diagnosed with hypothyroidism for the first time at presentation (17). They identified respiratory failure, coagulopathy, sepsis, and upper gastrointestinal bleeding as predictors of mortality in these patients (17). Hypothyroidism patients with elevated TSH levels may also exhibit AI (18), cardiac dysfunction (19), renal injury (20), and neurological derangements (21).

In the present study, six propensity-matched (based on age, BMI, and Zulewski’s score) patients without AI had significantly higher mean serum T3 and T4 levels than those with AI. Further, the serum cortisol levels of the case group were significantly lower than those of controls. Dutta et al. observed glucocorticoid deficiency in seven (30.4%) of 23 patients with myxedema coma (17). Moreover, Rodríguez-Gutiérrez et al. reported a 6.7% - 18.3% incidence of AI in patients with different degrees of primary hypothyroidism (18). Yamamoto identified comorbid latent primary AI in eight (5%) of 159 patients with autoimmune thyroid disease (hyperthyroidism and hypothyroidism) (22), whereas Ho et al. observed a 12% incidence of simultaneous hypothyroidism and AI in critically ill patients admitted to the intensive care unit (23). These findings indicate that AI is not uncommon among patients with severe hypothyroidism. Although the exact pathogenesis of this condition is unknown, evidence indicates the role of altered cortisol metabolism in the liver, inadequate adrenal response to the adrenocorticotrophic hormone, and altered pituitary function (7, 24).

However, in contrast to our findings, Seck et al. observed high cortisol values in 12.5% of patients with hypothyroidism, which they attributed to an increased half-life of cortisol and decreased metabolic clearance (25). Alternatively, patients with AI have also been found to exhibit low-to-normal T4 levels and elevated TSH levels, which further supports the coexistence of primary hypothyroidism and AI (26, 27). Data from a Norwegian registry showed that 41% of patients with primary AI had co-existing hypothyroidism (28). Several potential factors may explain the elevation of TSH levels in AI: Coexistence of primary hypothyroidism alongside primary AI, reduced responsiveness of the thyroid gland to TSH in hypocortisolemia, and altered metabolism in response to lower thyroid hormone production (29).

Adrenal insufficiency in patients with hypothyroidism may be transient and may improve with the treatment of hypothyroidism. Rodríguez-Gutiérrez et al. observed normal cortisol levels following levothyroxine initiation in approximately 81% of primary hypothyroidism patients with AI (18). Therefore, assessing the adrenal function of patients with severe hypothyroidism is essential. Immediate initiation of steroids may not be necessary upon detecting AI, as it may be transient and may reverse with thyroid hormone replacement. Similarly, TSH elevation secondary to AI may reverse after glucocorticoid replacement (27, 29, 30). The TSH levels of patients may oscillate during steroid therapy without changes in the thyroid hormone levels (27). Patients who do not recover normal cortisol levels after levothyroxine therapy should be monitored. Further, response to the cosyntropin test may normalize after thyroxine replacement (18).

To our knowledge, this is one of the first studies from the Indian subcontinent to assess adrenal function in a relatively large sample of patients with severe hypothyroidism (n = 71), which is the primary strength of our study. Nevertheless, this study is not without limitations. First, we could not assess antibodies, specifically anti-thyroid peroxidase and anti-adrenal antibodies, due to financial constraints. However, the presence or absence of thyroid antibodies would not have influenced our treatment decision. Second, determining the impact of thyroid replacement on AI was beyond the scope of this study. In addition, although the short synacthen test is optimal for adrenal function evaluation, it is currently considered a research tool until further investigations underscore its relevance in routine clinical practice with TSH >100 mIU/mL at diagnosis. Further tests, including those for adrenocorticotropic hormone stimulation, dehydroepiandrosterone levels, and plasma aldosterone and renin activity, could not be performed in the absence of specific clinical symptoms and indications owing to resource-constrained settings.

In this prospective study, we observed potential AI in 8.5% of treatment-naïve patients with severe hypothyroidism at first presentation. Clinical diagnosis of adrenal dysfunction in such cases is challenging. Therefore, clinicians must be vigilant and promptly identify any feature of AI after initiating levothyroxine, especially in the first week. In addition, adrenal function must be assessed upon diagnosis of severe hypothyroidism to exclude AI. Testing of morning serum cortisol levels is a standard and cost-effective first step for diagnosing AI (31, 32). The present findings may be further validated through a comprehensive study of patients with TSH levels of > 100 mIU/mL using the short synacthen test.

### 5.1. Limitations

The absence of ACTH (cosyntropin) stimulation testing limits the definitive diagnosis of AI. While basal morning serum cortisol measurements provide a screening estimate, they cannot definitively distinguish between functional hypothalamic-pituitary-adrenal axis suppression and true primary or secondary AI. The study did not evaluate anti-thyroid peroxidase or anti-adrenal antibodies, which could have provided insight into autoimmune etiologies of adrenal and thyroid dysfunction. This was a single-center study with only six patients meeting the criteria for AI, which limits the generalizability of findings and statistical power for subgroup analysis. Other useful tests, such as plasma ACTH, DHEA-S, renin, and aldosterone, were not performed due to logistical and financial limitations in the resource-constrained setting.

## Data Availability

The datasets generated during and/or analyzed during the current study are available from the corresponding author on reasonable request.
